# A Case of Dermoid Cyst of the Parotid Gland, with Specimen*Read before the Homœopathic Medical Society of the County of New York, November 9th, 1893.

**Published:** 1894-01

**Authors:** Edmund Carleton

**Affiliations:** New York


### A CASE OF DERMOID CYST OF THE PAROTID GLAND, WITH SPECIMEN.*

EDMUND CARLETON, M. D., NEW YORK.

This specimen was taken from the region of the right parotid gland, in the case of a young married lady, the mother of two children, and patient of our colleague, Dr. A. C. R. Stevens, October 12th, 1893. I am glad that the Committee on Pathology is to report also this evening, as the case should come to the notice of that committee equally with that of surgery.

A few years ago a physician removed a hard tumor from the same spot. At the time he told the family that it was a fibroid tumor; whether or not after minute examination is unknown to me. Antiseptics were used. Erysipelas followed, with slow convalescence and recovery.

About a year ago, patient discovered a hard swelling, which steadily grew to the proportions you now observe. Though bound down firmly at its base, it was less adherent at the sides.

---

*Read before the Homœopathic Medical Society of the County of New York, November 9th, 1893.

Removal was effected while the patient was under the influence of Nitrous Oxide Gas, given by the dentist, Dr. F. Hasbrouck. This seemed to me the best plan, for obvious reasons; and numerous trials of it have invariably given satisfaction. There are but a mouth-piece and a tube in the way, allowing ample room for work; it is a cleanly process, without sign of emesis; it is safe; time unrestricted.

Dissection was necessarily careful; but by keeping close to the tumor and using the edge of the knife but little, all accidents were avoided. A fragment of the tumor about the size of a filbert was torn from the main portion and somehow lost; otherwise it is all here in one mass.

I am sorry to dissapoint our antiseptic friends who are looking for more bacilli to conquer; but truth compels me to admit, that simple cleanliness only was relied upon during the operation and with the subsequent dressing. After the tumor had been removed the cavity was sponged clean with Calendula and water. A few threads of silk were laid in, to facilitate drainage; the edges fastened together with silk stitches; a compress of cotton and cheese-cloth applied; and slight, uniform pressure maintained with a roller bandage. On the fourth day, when the dressings were removed, all the parts were healed, excepting only where threads prevented. I have yet to be convinced, that simple, homœopathic methods, in medicine and in surgery, are not the best.

Our colleagues, Drs. Stevens, Fralick, Dyer, and O'Brien, kindly assisted me during the operation.

Examination shows that the growth is a dermoid cyst. The pathologist, Dr. C. Heitzman, under date of October 15th, certifies as follows: "The tumor, brought for examination last Friday, after hardening in a dilute chromic acid solution, under the microscope exhibited the following features:

"The central crumbly whitish mass consists of epidermal scales, partly in fatty degeneration, filling a small cystic cavity. The wall of the cyst has a few red-brown specks, which are remnants of blood. The thick capsule of the cyst consists of a dense fibrous connective tissue, in which there are imbedded numerous, mostly compressed, acini of the parotid gland. Nowhere was any inflammatory infiltration visible.

"*Diagnosis:* Dermoid cyst, probably an original cholesteatome, of the parotid gland; due to an incomplete obliteration of a branchial duct, in the earliest stages of embryoneal development. The tumor is entirely benign."

Believing the case to be sufficiently uncommon to make it interesting, I have brought it to your notice.

### Discussion.

Dr. F. E. Doughty spoke for those doctors who went "gunning for microbes." They used antiseptics to ease their consciences. As a matter of fact, however, wounds healed without antiseptics. He remembered a compound fracture of the thumb that recovered perfectly, notwithstanding the unfavorable condition it was in when first seen. It was washed clean, and then received simple dressings only.

Dr. T. M. Dillingham understood that Dr. Carleton had never adopted the antiseptic plan, but had always urged simple cleanliness and pure Homœopathy. After ample trial, many of the old school had lost faith in antiseptics; especially so after Tait's demonstrations in a very large number of cases, so that now the profession are coming to accept the views which Dr. Carleton has advocated all the time.

